# The γ-Protocadherin-C3 isoform inhibits canonical Wnt signalling by binding to and stabilizing Axin1 at the membrane

**DOI:** 10.1038/srep31665

**Published:** 2016-08-17

**Authors:** Kar Men Mah, Douglas W. Houston, Joshua A. Weiner

**Affiliations:** 1 Department of Biology, The University of Iowa, 143 Biology Building, Iowa City, 52242, IA, USA; 2 Integrated Biology Graduate Program, The University of Iowa, 143 Biology Building, Iowa City,52242, IA, USA; 3 Department of Psychiatry, University of Iowa Carver College of Medicine, 200 Hawkins Drive, Iowa City, 52242, IA, USA

## Abstract

The 22 γ-Protocadherin (γ-Pcdh) adhesion molecules encoded by the *Pcdhg* gene cluster play critical roles in nervous system development, including regulation of dendrite arborisation, neuronal survival, and synaptogenesis. Recently, they have been implicated in suppression of tumour cell growth by inhibition of canonical Wnt signalling, though the mechanisms through which this occurs remain unknown. Here, we show differential regulation of Wnt signalling by individual γ-Pcdhs: The C3 isoform uniquely inhibits the pathway, whilst 13 other isoforms upregulate signalling. Focusing on the C3 isoform, we show that its unique variable cytoplasmic domain (VCD) is the critical one for Wnt pathway inhibition. γ-Pcdh-C3, but not other isoforms, physically interacts with Axin1, a key component of the canonical Wnt pathway. The C3 VCD competes with Dishevelled for binding to the DIX domain of Axin1, which stabilizes Axin1 at the membrane and leads to reduced phosphorylation of Wnt co-receptor Lrp6. Finally, we present evidence that Wnt pathway activity can be modulated up (by γ-Pcdh-A1) or down (by γ-Pcdh-C3) in the cerebral cortex *in vivo*, using conditional transgenic alleles. Together, these data delineate opposing roles for γ-Pcdh isoforms in regulating Wnt signalling and identify Axin1 as a novel protein interactor of the widely-expressed γ-Pcdh-C3 isoform.

Protocadherins (Pcdhs) belong to the cadherin superfamily, a group of cell adhesion molecules that are known to play critical roles in several biological processes, including embryonic morphogenesis, neural circuit formation, angiogenesis, and cancer^1^,^2^,^3^. The clustered Pcdhs, consisting of ~60 proteins encoded by three tandem gene clusters (*Pcdha, Pcdhb*, and *Pcdhg*) on human chromosome 5q31 (chromosome 18 in the mouse), represent the largest subgroup within the cadherin superfamily^4^,^5^. Within the *Pcdha* and *Pcdhg* clusters, large “variable” exons encoding 6 extracellular cadherin (EC) repeats, a transmembrane domain, and a variable cytoplasmic domain (VCD) are expressed from their own promoters and spliced to three short constant exons that encode a C-terminal domain shared by all α- or γ-Pcdhs (β-Pcdhs do not have this shared domain and are thus each encoded by a single exon; *Pcdhg* cluster schematized in Fig. 1a)^4^,^6^,^7^. Clustered Pcdhs, as a group, are strongly expressed throughout the developing and mature nervous system^8^,^9^,^10^,^11^,^12^, with lower levels detected in other organs such as lung and kidney^13^,^14^. The expression of individual clustered Pcdh genes is differentially regulated in each cell via promoter methylation and interaction with distant regulatory elements^15^,^16^,^17^,^18^. Single-cell RT-PCR analysis suggests that each neuron expresses a fraction of the 14 *Pcdha*, 22 *Pcdhb*, and 22 *Pcdhg* genes; most are stochastically and sparsely expressed, though 5 “C” subtype genes (*Pcdhac1, Pcdhac2, Pcdhgc3, Pcdhgc4,* and *Pcdhgc5*) appear to be expressed ubiquitously^19^,^20^,^21^. All clustered Pcdhs form *cis-*multimers that interact in a strictly homophilic manner in *trans*^22^,^23^,^24^.

The clustered Pcdhs play critical roles in neurodevelopment. Using constitutive and conditional *Pcdhg* mouse mutants, we demonstrated that the γ-Pcdhs regulate neuronal survival^11^,^25^, synaptogenesis^26^,^27^, astrocyte-neuron interactions^26^, dendrite arborisation^8^,^28^,^29^, and axonal patterning^30^. In retinal starburst amacrine cells, the γ-Pcdhs are also required for normal dendrite self-avoidance^31^,^32^, underscoring the importance of this family for the proper formation of dendrite arbours. The relative importance of the γ-Pcdhs is underscored by the fact that mice lacking all 22 isoforms die shortly after birth^7^, in contrast to the viable and fertile *Pcdha* cluster nulls^33^. Nevertheless, the α-Pcdhs have been shown to regulate dendrite arborisation and dendritic spine formation^34^, as well as axonal targeting^33^,^35^,^36^,^37^, in a variety of neurons.

Intriguingly, the γ-Pcdhs also have been recently implicated as potential tumour suppressors, via inhibition of canonical Wnt signalling^13^. A genome-wide analysis of promoter hypermethylation in Wilms’ tumour, a paediatric kidney cancer, identified the clustered Pcdh genes as consistently hypermethylated; many of them, especially those encoding γ-Pcdhs, are indeed transcriptionally silenced in tumour cells. Knockdown of all *Pcdhg* genes (using an siRNA targeting the constant region) in kidney cell lines led to increased canonical Wnt signalling, while overexpression of individual *Pcdhg* cDNAs inhibited the Wnt pathway and led to reduced tumour cell growth in soft agar assays^13^. The ubiquitously-expressed γ-Pcdh-C3 isoform (encoded by *Pcdhgc3*) was similarly shown to be silenced in colorectal cancer cells, and to reduce growth of these cells when overexpressed via inhibition of a Wnt-mTOR pathway^38^. Together with other studies identifying aberrant methylation of *Pcdhga11* in astrocytomas^39^, *Pcdhb* genes in neuroblastoma^40^, and *Pcdhg* genes in toxicant-induced malignant cells^41^, these data suggest that understanding how clustered Pcdhs regulate tumour growth pathways will be of future translational importance. Intriguingly, a non-clustered δ-Pcdh known as PAPC/Pcdh8/Arcadlin can also affect canonical^42^ and non-canonical^43^,^44^ Wnt pathways, suggesting that Pcdhs in general may be of interest to our understanding of Wnt biology.

Here, we have investigated the molecular mechanisms through which γ-Pcdh-C3 inhibits canonical Wnt signalling, a well-known tumourigenic pathway that is also of paramount importance for many steps in neurodevelopment^45^,^46^. Binding of Wnt ligands to Frizzled and the Lrp5/6, Ryk, or ROR co-receptors can activate at least three distinct pathways: the canonical (β-catenin/TCF), PCP (planar cell polarity), and Wnt-Ca2 + pathways^47^,^48^,^49^. In the canonical pathway, β-catenin is found in a complex including Axin1, Adenomatous Polyposis Coli (APC) and GSK3β in the absence of Wnt ligand (the OFF state; reviewed in Clevers and Nusse, 2012)^50^. Axin1 acts as a scaffold for the “destruction complex”: GSK3β phosphorylates β-catenin, which is then targeted for ubiquitin-dependent degradation by the proteasome. In the presence of the Wnt ligand (the ON state), Dishevelled (Dvl) binds at the C-terminus of Frizzled^51^ and recruits Axin1 to the cytoplasmic tail of Lrp5/6, facilitating the phosphorylation of Lrp5/6 by GSK3β and CK1^52^,^53^,^54^,^55^,^56^,^57^. Phospho-Lrp6 is thought to directly inhibit GSK3β activity in the destruction complex, leading to reduced phosphorylation of β-catenin as well as Axin1 itself. Dephosphorylated Axin1 dissociates from the activated receptor complex and also from β-catenin, thus inactivating the destruction complex until Axin1 is again phosphorylated^55^,^58^. This inactivation of the destruction complex is thought to allow β-catenin to accumulate in the cytoplasm and to translocate to the nucleus. There, it displaces Groucho/TLE corepressors bound to TCF (T-cell factor)/LEF (Lymphoid enhancer factor) transcription factors, thereby activating Wnt target genes, including many that promote tumour proliferation or regulate brain patterning, dendrite arborisation, and synaptogenesis^45^,^50^,^59^.

Though the γ-Pcdhs have been implicated in the regulation of Wnt signalling^13^,^38^, the underlying molecular mechanisms are unknown. Here, we show that γ-Pcdh-C3, but not other γ-Pcdhs, significantly inhibits exogenous activation of canonical Wnt signalling in cultured cells, and identify the C3-specific VCD as the critical site of action. Though it has been reported that the intracellular domain of γ-Pcdhs can be cleaved and trafficked to the nucleus^60^,^61^, we show that the C3 VCD acts at the membrane, not the nucleus, to inhibit Wnt signalling. We further show that the C3 VCD physically, and directly, interacts with the DIX domain of Axin1, and present evidence that it competes for this binding site with Dvl1. Binding of Axin1 to the C3 VCD stabilizes Axin1 at the membrane and prevents Lrp6 phosphorylation, suggesting that in this context, membrane localization can actually stabilize the β-catenin destruction complex. Finally, we use a Wnt signalling reporter mouse line and an inducible γ-Pcdh-C3 overexpression allele to show that that increasing C3 levels reduces endogenous Wnt signalling in the mouse cerebral cortex *in vivo*. Together, these data identify a novel molecular mechanism by which γ-Pcdhs can affect Wnt signalling, and identify Axin1 as the first known intracellular signalling partner for the widely-expressed γ-Pcdh-C3 isoform.

## Results

### γ-Pcdh isoforms differentially regulate canonical Wnt signalling

**Figure 1:**
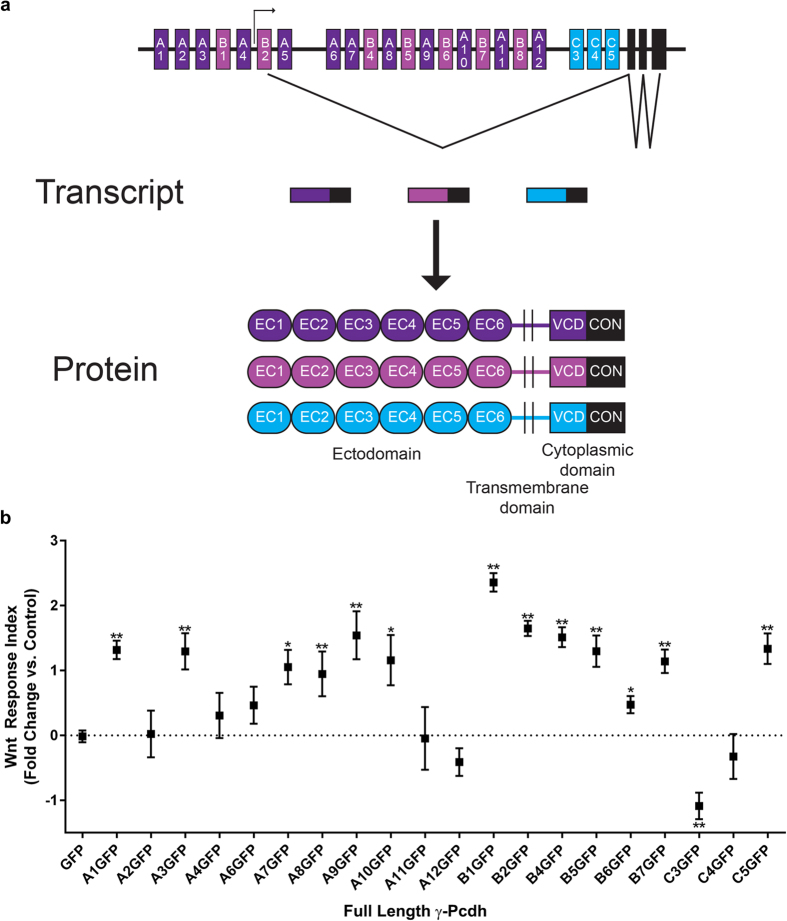
Differential regulation of canonical Wnt signalling by individual γ-Pcdh isoforms. (**a**) Schematic of the mouse *Pcdhg* gene cluster (top), with examples of promoter activation, gene transcription, and splicing. Example transcripts and resultant proteins are schematized below. Each of the 22 isoforms is encoded by 4 exons: 1 variable exon (shades of purple or blue; encodes 6 EC domains, a transmembrane domain, and a VCD) and 3 constant exons (black; encode a shared C-terminal domain). EC: extracellular cadherin repeats; VCD: variable cytoplasmic domain; CON: constant region. (**b**) TOPFlash assays were performed by co-transfecting plasmids encoding individual γ-Pcdh isoforms with the SUPER-TOPFlash and *Renilla* luciferase plasmids into HEK293 cells. Cell lysates were assayed for luciferase activity 24 hours after exposure to Wnt3a CM. Results are presented as a “Wnt Response Index”, defined as the fold change in Relative Luciferase Units (RLU; ratio of firefly luciferase to *Renilla* luciferase [control for transfection efficiency]) following Wnt treatment in cells transfected with individual γ-Pcdh isoforms compared to that in cells expressing only a GFP negative control. Thus, the control (GFP only) Wnt cellular response is the baseline (“0”), and manipulations that increase further that response yield a positive index, while those that decrease that response yield a negative index. Several γ-Pcdh isoforms significantly upregulated Wnt signalling activity, while only γ-Pcdh-C3 significantly downregulated Wnt signalling. Data are presented on a log2 scale of means ± SEM from 6 independent experiments. A two-way ANOVA with Bonferroni post hoc test (to correct for multiple comparisons) was performed to assess statistical significance. *p < 0.05, and **p < 0.01.

We began by asking whether the many γ-Pcdh isoforms (Fig. 1a) uniformly, or differentially, affect the Wnt signalling pathway. To assay Wnt activity, we utilized the well-characterized TOPFlash assay, in which a TCF/LEF-responsive promoter drives firefly luciferase, levels of which can be normalized to constitutively-expressed *Renilla* luciferase to control for transfection efficiency in HEK293 cells^62^. To activate canonical Wnt signalling, we applied media conditioned by L cells stably expressing Wnt3a^63^. Dallosso *et al*.^13^,^38^ previously reported that human γ-Pcdh-A2, -A6, -A12 and -C3 were all capable of inhibiting aberrantly high endogenous TOPFlash signal in Wilms’ tumour cell lines, or the increase seen following Wnt treatment in HEK293 cells. We individually overexpressed 20 γ-Pcdh isoforms in HEK293 cells and found differential activity among γ-Pcdhs. In agreement with Dallosso *et al*.^38^, overexpression of the C3 isoform reliably and significantly inhibited Wnt signalling (Fig. 1b), though in contrast to Dallosso *et al*.^13^ we found that A2, A6, or A12 overexpression had no significant effect (Fig. 1b). Surprisingly, we also found that overexpression of several other isoforms, including A1, A3, A7-10, B1-7, and C5, all significantly *increased* the TOPFlash response in HEK293 cells (Fig. 1b). Results are presented throughout as a “Wnt Response Index”, defined as the fold change the Wnt response in cells overexpressing a γ-Pcdh compared to that in cells expressing only a GFP negative control. Example raw TOPFlash and FOPFlash (negative control plasmids in which TCF/LEF sites are mutated) data are presented in Supplementary Figure S1; FOPFlash results were always essentially zero and are not shown for subsequent experiments. In all subsequent analyses, we focus on the C3 isoform because: 1) our results in this case are fully in agreement with those of Dallosso *et al*.^38^ in other cell lines; 2) C3 is the only isoform that significantly downregulated Wnt signalling; and 3) C3 is highly and ubiquitously expressed and thus is likely to be of importance in the greatest number of contexts.

### The variable cytoplasmic domain of γ-Pcdh-C3 mediates inhibition of Wnt signalling

**Figure 2:**
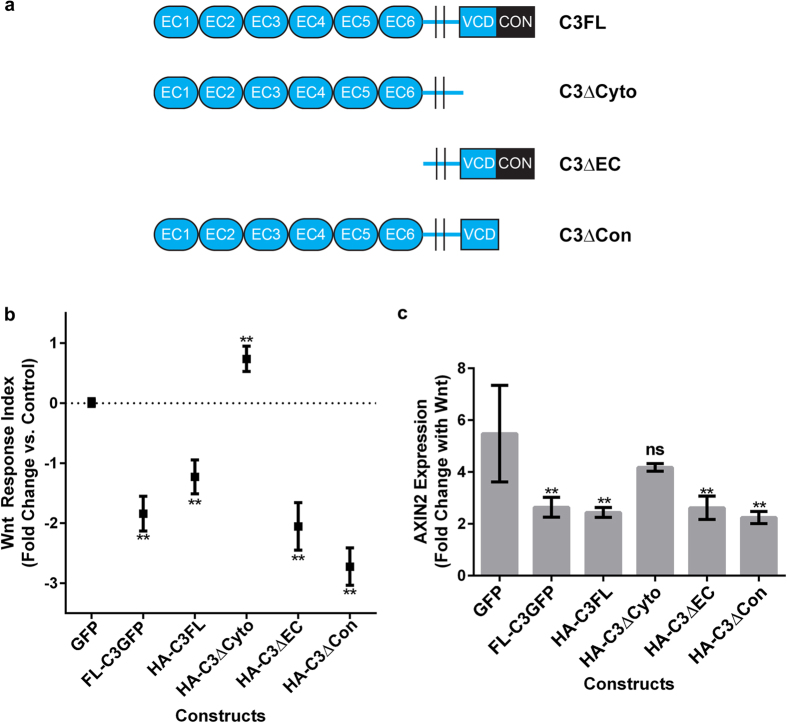
The variable cytoplasmic domain (VCD) is critical for γ-Pcdh-C3 inhibition of Wnt signalling. (**a**) Schematic of constructs used in experiments to determine the domain involved in γ-Pcdh-C3’s regulation of Wnt signalling. All are HA-tagged at the N-terminus. (**b**) The aforementioned constructs were used in a TOPFlash assay, as previously described. All constructs except HA-C3ΔCyto significantly inhibited Wnt signalling response 24 hours after the addition of Wnt3a CM, implicating the VCD as the key domain. Data are presented on a log2 scale of means ± SEM from 6 independent experiments. A two-way ANOVA with Bonferroni post hoc test (to correct for multiple comparisons) was performed to assess statistical significance. **p < 0.01 (**c**) Quantitative TaqMan® PCR on *AXIN2,* a Wnt target gene, was performed 24 hours after exposure to Wnt3a CM in HEK293 cells transfected with the constructs in (**a**). Data are presented as fold increase in compared to DMEM media change control for cells transfected with each construct. *AXIN2* expression increases ~5-fold in the GFP transfected control, 24 hours after addition of Wnt3a CM. This increase is significantly abrogated in cells expressing all γ-Pcdh-C3 constructs except HA-C3ΔCyto, paralleling TOPFlash results in (**b**). Means ± SEM of *AXIN2* expression from 3 independent experiments were graphed. A two-way ANOVA with Bonferroni post hoc test (to correct for multiple comparisons) was performed to assess statistical significance. **p < 0.01.

We next asked which domain of the γ-Pcdh-C3 protein was important for its inhibition of Wnt signalling. As noted above, each γ-Pcdh isoform is encoded by four exons: the large variable exon, specific to each isoform, encodes 6 EC repeats (EC2 and 3 determine homophilic binding specificity^23^;), a transmembrane domain, and the ~90 amino acid VCD; the 3 small constant exons encode a C-terminal domain shared by all γ-Pcdhs (Fig. 1a). We generated and tested the following constructs: γ-Pcdh-C3 ΔEC (lacking all 6 EC repeats); γ-Pcdh-C3 ΔCyto (lacking the entire intracellular domain, including both the VCD and the constant domain); and γ-Pcdh-C3 ΔCon (lacking only the shared constant domain) (Fig. 2a). When these constructs were used in the TOPFlash assay as described above, we found that all except the C3 ΔCyto construct were still able to negatively regulate Wnt signalling activity. Expression of C3 ΔCyto failed to inhibit Wnt activity, and in fact resulted in a small, but statistically significant, increase (Fig. 2b). To confirm these results, we utilized another assay for canonical Wnt signalling, performing quantitative RT-PCR for *AXIN2*, a Wnt target gene. In control (GFP-transfected) cells exposed to Wnt3a, *AXIN2* transcript levels increased more than five-fold (Fig. 2c). Overexpression of C3 full length (FL; either C-terminally GFP-tagged or N-terminally HA-tagged), C3 ΔEC, or C3 ΔCon significantly abrogated this *AXIN2* response, while overexpression of C3 ΔCyto did not (Fig. 2c). Together, these data implicate the C3 VCD as the protein domain responsible for inhibiting Wnt signalling.

### The variable cytoplasmic domain of γ-Pcdh-C3 acts at the membrane to inhibit Wnt signalling

**Figure 3:**
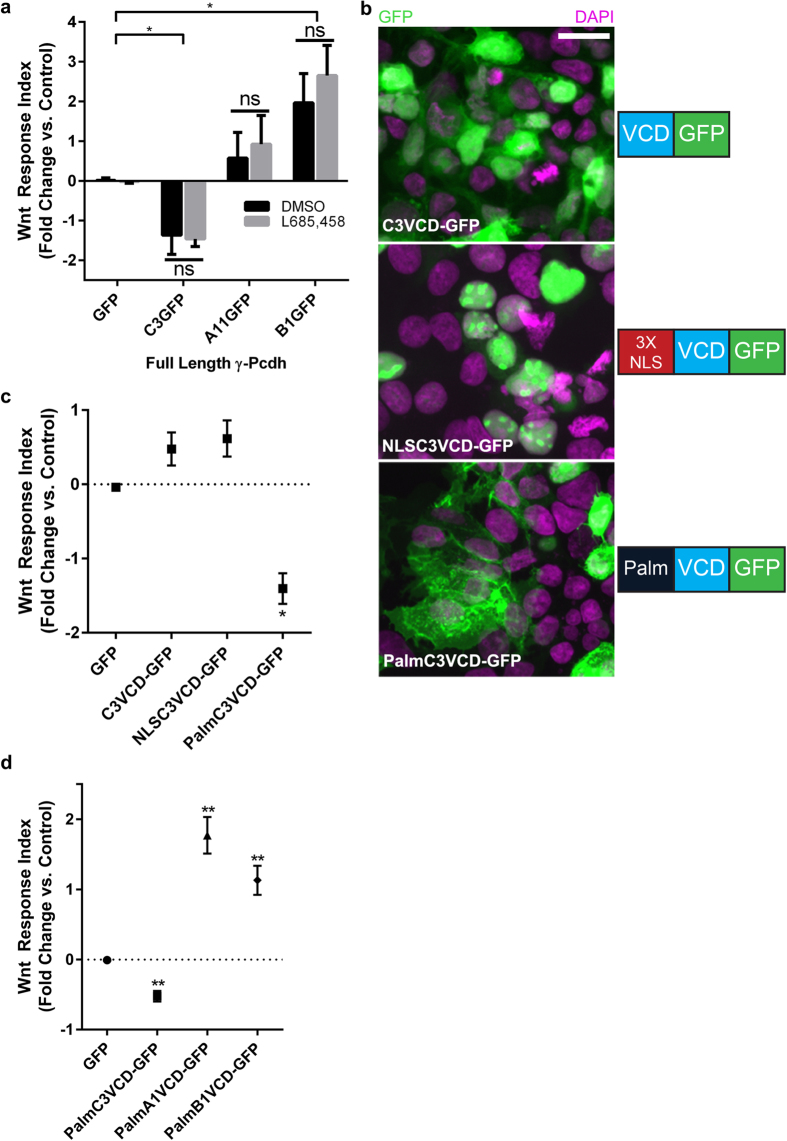
The γ-Pcdh-C3 VCD acts at the membrane to inhibit Wnt signalling. (**a**) Cells transfected with FL-C3GFP, FL-A11GFP and FL-B1GFP were treated with 10 μM L685,458 (to inhibit γ-secretase), or DMSO (vehicle control) and used in a TOPFlash assay, as previously described. Treatment with L685,458 did not significantly alter individual γ-Pcdh isoforms’ effect on Wnt signalling activity, indicating that C-terminal cleavage is not required. (**b**) Constructs encoding the isolated VCD of γ-Pcdh-C3 fused to GFP, either unmodified (C3VCD-GFP), tagged with a triplicate nuclear localization signal (NLSC3VCD-GFP) or with a palmitoylation sequence (PalmC3VCD-GFP) were generated. HEK293 cells transfected with these constructs were immunostained for GFP (green) and counterstained with DAPI (purple) to assess subcellular localization and visualize nuclei, respectively. Localization was as predicted: C3VCD-GFP localizes to both cytoplasm and nucleus, NLSC3VCD-GFP is restricted to nuclei, and PalmC3VCD-GFP is concentrated at the cell surface and at cell-cell junctions. Scale bar = 20 μm. (**c**) These new constructs were used in a TOPFlash assay, as described previously. PalmC3VCD-GFP was the only construct that significantly inhibited Wnt signalling activity; C3VCD-GFP and NLSC3VCD-GFP did not differ significantly from control. (**d**) Palmitoylated A1VCD-GFP and B1VCD-GFP were made and used in a TOPFlash assay. The VCD alone of these individual γ-Pcdh isoforms are sufficient to significantly upregulate Wnt signalling activity. Data are presented on a log2 scale of means ± SEM from 6 independent experiments. A two-way ANOVA with Bonferroni post hoc test (to correct for multiple comparisons) was performed to assess statistical significance. *p < 0.05 ** p < 0.01.

It has been reported that γ-Pcdhs can undergo two cleavage events: ectodomain shedding mediated by matrix metalloproteinases (MMPs) at the junction between the EC repeats and transmembrane domain, and cleavage at the junction between the intracellular and transmembrane domain by γ-secretase^60^,^61^. The cleaved intracellular domain (containing both the VCD and the constant domain) can translocate to the nucleus^60^,^61^, where we reasoned it might interfere directly with TCF/LEF-dependent Wnt target gene activation. We thus asked whether inhibition of γ-secretase cleavage would abrogate γ-Pcdh-C3’s ability to inhibit Wnt signalling. We treated cells with a specific pharmacological inhibitor of γ-secretase (L685,458), previously shown to block γ-Pcdh intracellular domain cleavage^60^,^61^. In the TOPFlash assay, there was no effect of treatment with L685,458 vs. the DMSO vehicle control: C3 significantly inhibited Wnt signalling, A11 had no effect, and B1 significantly potentiated Wnt signalling, as observed previously (Fig. 1b), regardless of inhibitor treatment (Fig. 3a). Western blots for the relevant C-terminal fragment confirmed the efficacy of L685,458 in this assay (Supplementary Figure S2). These observations indicate that γ-Pcdh C-terminal cleavage is not important for effects on Wnt signalling, and argue against a nuclear site of action for the C3 VCD.

To confirm this directly, we designed new constructs to investigate the cellular compartment at which the C3 VCD acts to inhibit Wnt signalling. These constructs encoded the isolated C3 VCD fused to GFP, either alone (C3VCD-GFP) or with the addition of a 3X nuclear localization signal (NLSC3VCD-GFP) or a membrane-targeting palmitoylation signal (PalmC3VCD-GFP). Each construct exhibited the expected subcellular localization when expressed in HEK293 cells (Fig. 3b). Of the three constructs, only the membrane-targeted palmitoylated version significantly inhibited Wnt signalling (assayed using TOPFlash) following addition of Wnt3a CM (Fig. 3c). These data indicate that the VCD of γ-Pcdh-C3 acts at the plasma membrane to regulate Wnt signalling, consistent with the normal topology of the full-length protein and with our demonstration that preventing cleavage of the C3 intracellular domain (Fig. 3a) did not disrupt Wnt inhibition. Given these results, we asked whether γ-Pcdh isoforms that upregulate Wnt signalling (Fig. 1b) also do so *via* their VCDs. We constructed plasmids encoding PalmA1VCD-GFP and PalmB1VCD-GFP and tested them in the TOPFlash assay as above. Indeed, we found that cells expressing either construct exhibited significantly enhanced response to Wnt3a (Fig. 3d), just as cells expressing their full length counterparts did (Fig. 1b).

### γ-Pcdh-C3 physically interacts with the DIX domain of Axin1 via its variable cytoplasmic domain

**Figure 4:**
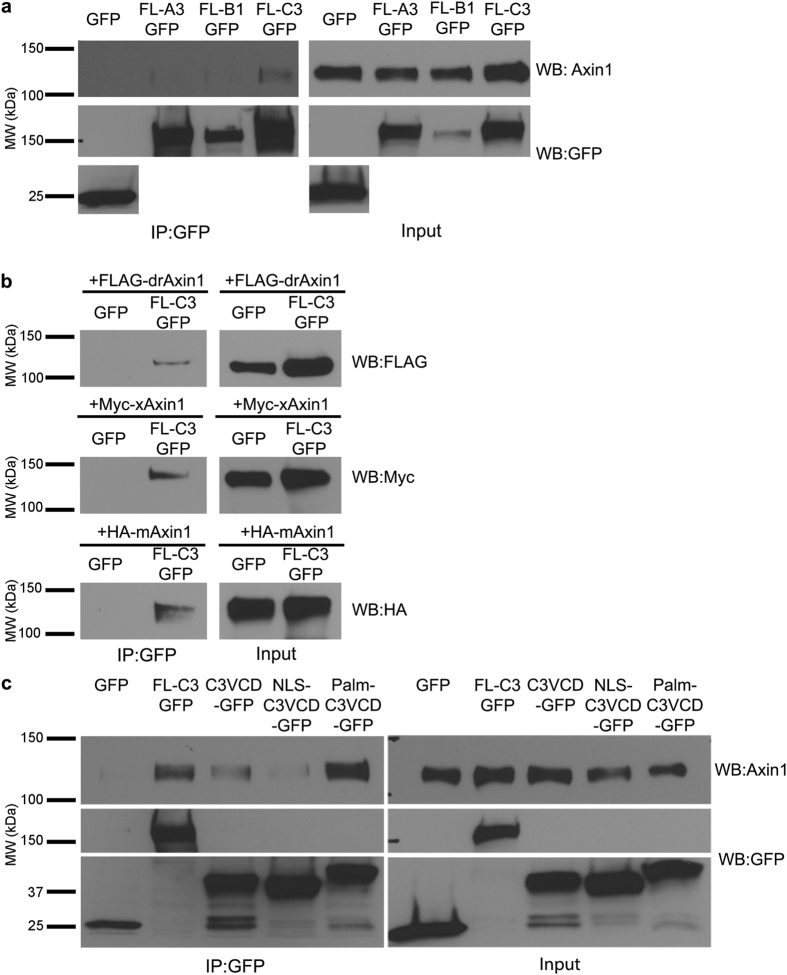
The γ-Pcdh-C3 VCD interacts with Axin1. (**a**) Lysates of HEK293 cells transfected with either GFP (control), FL-A3GFP, FL-B1GFP or FL-C3GFP were immunoprecipitated with anti-GFP and blotted for endogenous (human) Axin1. Axin1 co-immunoprecipitated with γ-Pcdh-C3 but not –A3 or –B1. All proteins encoded by the transfected constructs were present in the cell lysates used (right; input) and re-probing of blots with anti-GFP antibody confirmed immunoprecipitation. (**b**) Lysates from HEK293 cells co-transfected with GFP or FLC3-GFP and either FLAG-tagged zebrafish (*Danio rerio)* Axin1 (FLAG-drAxin1), Myc-tagged *Xenopus* Axin1 (Myc-xAxin1) or HA-tagged mouse Axin1 (HA-mAxin1) were immunoprecipitated with anti-GFP and blotted for the tagged Axin1. FL-C3GFP co-immunoprecipitated all three Axin1 orthologues. (**c**) Co-immunoprecipitation of endogenous HEK293 Axin1 as in (**a**) was performed using GFP, FLC3-GFP, C3VCD-GFP, NLSC3VCD-GFP and PalmC3VCD-GFP. In all cases, the VCD is sufficient to pull down endogenous Axin1; co-immunoprecipitation was more robust for PalmC3VCD-GFP. Blots shown are representative of at least 3 experiments. MW, molecular weight; kDa, kilodaltons.

Given that the C3 VCD inhibits Wnt signalling by acting at the plasma membrane, we hypothesized that C3 may physically interact with one of the many components of the Wnt pathway that can localize there during signalling. A previous large-scale proteomics study suggested that Ryk could be a potential target of some γ-Pcdhs, including C3^64^. We transfected HEK293 cells with plasmids encoding FL C3-GFP, or GFP only as a negative control, immunoprecipitated using anti-GFP antibody, and blotted with antibodies to a variety of Wnt pathway proteins including Ryk, Dvls, Frizzleds, Lrp6, Naked, GSK3β, and Axin1. The only endogenous human protein that co-immunoprecipitated with mouse FL C3-GFP was Axin1 (Fig. 4a; we were unable to confirm an interaction with Ryk^64^ despite multiple attempts). We were unable to co-immunoprecipitate Axin1 reliably with two other γ-Pcdhs tested (A3 or B1), suggesting that this interaction is specific to γ-Pcdh-C3 (Fig. 4a). Using a variety of tagged Axin1 plasmids in a co-transfection immunoprecipitation assay, we found this interaction to be evolutionarily conserved, as mouse FL C3-GFP could pull down FLAG-tagged zebrafish (drAxin1), *Xenopus* (xAxin1), and mouse (mAxin1) proteins (Fig. 4b) as well as endogenous human Axin1 expressed by HEK293 cells. Given that we found that the VCD of C3 is the critical domain for Wnt pathway inhibition, we next tested whether Axin1 interacted with this domain in particular. We found that the C3 VCD co-immunoprecipitated endogenous Axin1; intriguingly, the PalmC3VCD construct consistently showed a more robust interaction with Axin1 than did the cytoplasmic or nuclear constructs (Fig. 4c). This result suggests that at least some of the C3VCD-Axin1 complex co-immunoprecipitated in this assay formed in the cell, at the membrane, rather than in solution following lysis.

**Figure 5:**
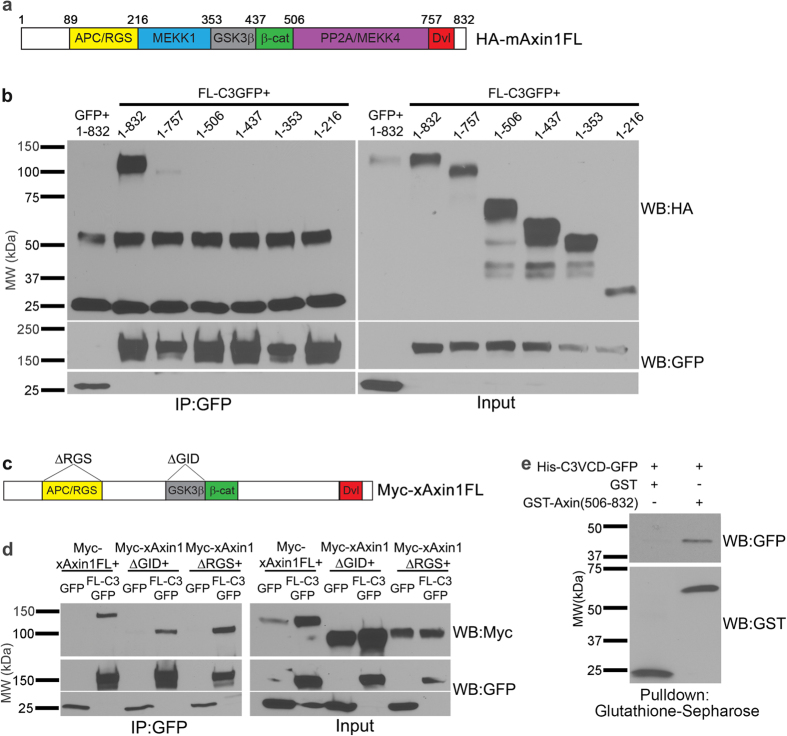
γ-Pcdh-C3 interacts with the DIX domain of Axin1. (**a**) Schematic of specific protein-protein interacting domains previously identified in mouse Axin1. Numbers above the protein correspond with the amino acid at which C-terminal truncations were made to sequentially remove individual domains. (**b**) Lysates from HEK293 cells co-transfected with GFP or FL-C3GFP and the HA-tagged full-length mouse Axin1 (1-832); or FL-C3GFP with HA-tagged mouse Axin1 with progressive 3′ deletions (containing amino acids 1-757, 1-506, 1-437, 1-353 or 1-216) were immunoprecipitated with anti-GFP and blotted for HA. Interaction of γ-Pcdh-C3 with Axin1 was abrogated with the loss of amino acids 758-832, indicating the importance of the DIX domain. (**c**) Schematic of Myc-tagged full length *Xenopus* Axin1 (Myc-xAxin1FL) and Myc-tagged *Xenopus* Axin1 with the APC/RGS binding domain (Myc-xAxinΔRGS) or the GSK3β binding domain deleted (Myc-xAxin1ΔGID). (**d**) Lysates from HEK293 cells co-transfected GFP or FL-C3GFP and Myc-xAxin1FL, Myc-xAxinΔRGS, or Myc-xAxin1ΔGID were immunoprecipitated with anti-GFP and blotted for Myc. Interaction between γ-Pcdh-C3 and Axin1 was observed with all three constructs used, confirming that these internal domains are not critical. (**e**) Purified His-tagged C3VCD-GFP was mixed with either GST alone, or with GST-Axin(506-832) in a direct binding assay. MW, molecular weight; kDa, kilodaltons.

We next sought to identify the domain of Axin1 to which the C3 VCD binds. The protein structure of Axin1 has been well studied, leading to the identification of multiple conserved domains and regions of protein-protein interactions. These include binding regions for APC (the RGS domain), MEKK1, GSK3β, β-catenin, PP2A/MEKK4, and Dvl (DIX domain; Fig. 5a). We utilized constructs^65^ encoding HA-tagged mAxin1 with progressive 3′ deletions leading to loss of these domains in co-immunoprecipitation assays with FL C3-GFP (Fig. 5b). Full length mAxin1 protein again exhibited robust interaction with γ-Pcdh-C3; this interaction was almost completely abrogated following the loss of amino acids 757-832, and no interaction was observed with further mAxin1 truncation (Fig. 5b). This result indicates that the C3 VCD interacts with mAxin1 via the latter’s C-terminal Dvl-binding DIX domain. However, since nearly all binding was lost with the very first 3′ truncation, we sought to confirm this by utilizing two Myc-tagged xAxin1 constructs with internal deletions of the GSK3β (ΔGID) or APC (ΔRGS) domains (Fig. 5c). As expected, both of these truncated constructs, as well as FL xAxin1, robustly interacted with FL C3-GFP in transfected HEK293 cells (Fig. 5d). Co-immunoprecipitation can reflect either direct binding between two proteins, or their mutual interaction with a complex of proteins. To ascertain whether the interaction between Axin1 and C3 VCD was direct, we generated new constructs for bacterial production of His-tagged C3VCD-GFP and GST-tagged mouse Axin1(506-832; encompassing the PP2A/MEKK4 and DIX domains), and assessed binding in a cell-free assay. Using glutathione-sepharose to pull down GST-fusion proteins, we recovered His-C3VCD-GFP with GST-Axin1(506–832), but not with GST only (Fig. 5e). Together, our data indicate a direct physical interaction between the VCD of γ-Pcdh-C3 and the DIX domain of Axin1.

### γ-Pcdh-C3 competes with Dvl1 for Axin1 binding and stabilizes Axin1 at the membrane

**Figure 6:**
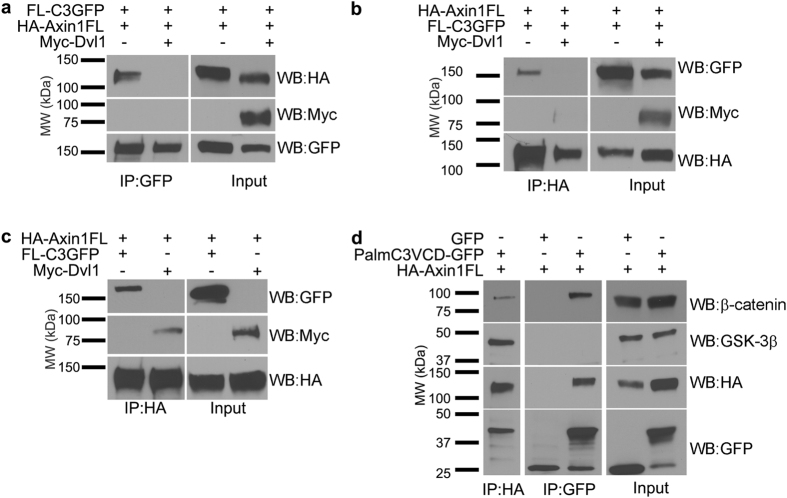
γ-Pcdh-C3 competes for Dvl1 binding to Axin1. (**a**,**b**) Lysates from HEK293 cells co-transfected with FL-C3GFP and HA-Axin1FL, with or without Myc-Dvl1 were immunoprecipitated with anti-GFP or anti-HA to pull down γ-Pcdh-C3 or Axin1, respectively. In either case, the interaction between γ-Pcdh-C3 and Axin1 is abrogated in the presence of Dvl1. (**c**) Lysates from HEK293 cells co-transfected with HA-Axin1FL and FL-C3GFP or Myc-Dvl1 were immunoprecipitated with anti-HA to pull down Axin1, and blotted for GFP to detect γ-Pcdh-C3 or Myc to detect Dvl1. Axin1 can co-immunoprecipitate either γ-Pcdh-C3 or Axin1, as long as both are not present. (**d**) HEK293 cells co-transfected with HA-Axin1FL and either GFP or PalmC3VCD-GFP were immunoprecipitated with anti-GFP or anti-HA, and blotted for endogenous β-catenin and GSK3β (components of the Wnt destruction complex). Axin1 interacts with both β-catenin and GSK3β, while γ-Pcdh C3 interacts with β-catenin, but apparently not with GSK3β. MW, molecular weight; kDa, kilodaltons.

As noted above, we observed that the Dvl-binding DIX domain of Axin1 was important for its interaction with γ-Pcdh-C3 (Fig. 5b). Thus, we next asked whether Dvl and γ-Pcdh-C3 might compete for Axin1 binding. To test this, HEK293 cells were co-transfected with constructs encoding FL C3-GFP and HA-tagged mAxin1, with or without the addition of a third plasmid encoding Myc-tagged Dvl1. We then immunoprecipitated with antibodies against GFP (for the γ-Pcdh-C3) and blotted for the HA-Axin1, or immunoprecipitated with antibodies against HA (for the Axin1) and blotted for the C3-GFP. These reciprocal immunoprecipitations both show that the interaction between γ-Pcdh-C3 and Axin1 is lost when Dvl1 is overexpressed (Fig. 6a,b). Interestingly, Dvl1 did not co-immunoprecipitate with Axin1 in the presence of γ-Pcdh-C3 (Fig. 6b), although it did, as expected, in its absence (Fig. 6c), indicating that C3 effectively competes with Dvl1 for Axin1 binding. We next asked if γ-Pcdh-C3 interacts with other destruction complex components to regulate Wnt signalling. We overexpressed PalmC3VCD-GFP and full length HA-tagged mAxin1 in HEK293 cells, immunoprecipitated with either anti-HA or anti-GFP, and blotted for endogenous β-catenin or GSK3β. A previous proteomics study^66^ recovered β-catenin in material pulled down from brain with an antibody recognizing all γ-Pcdhs. We were able to confirm an interaction of the C3VCD with β-catenin in these transfected HEK293 cells (Fig. 6d); note that this likely to be indirect *via* Axin1, as γ-Pcdhs (unlike classical cadherins) do not exhibit any known catenin binding sites. We also were able to co-immunoprecipitate both β-catenin and GSK3β with Axin1, as expected^67^. However, we were unable to observe an interaction between γ-Pcdh C3 and GSK3β in this assay (Fig. 6d). This suggests that γ-Pcdh-C3 can interact with Axin1 and β-catenin, but probably not in the context of a complete destruction complex.

**Figure 7:**
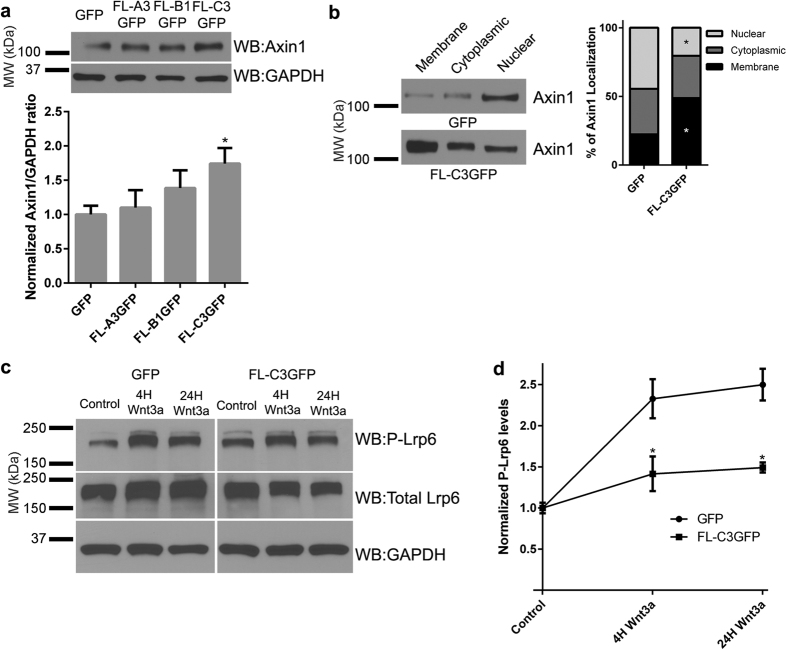
Interaction between γ-Pcdh C3 and Axin1 stabilizes Axin1 at the membrane and prevents phosphorylation of Lrp6. (**a**) Representative Western blot showing increased endogenous Axin1 levels in HEK293 cells overexpressing FL-C3GFP in comparison to GFP, FL-A3GFP, or FL-B1GFP. The band intensity of Axin1 was quantified and normalized to that of GAPDH in the same lane. Means ±  SEM of 4 independent experiments were graphed. A one-way ANOVA with Bonferroni post hoc test (to correct of multiple comparisons) was used to assess statistical significance. Only γ-Pcdh-C3 significantly increased levels of Axin1. *p < 0.05 (**b**) Subcellular fractionation of HEK293 cell lysates overexpressing FL-C3GFP shows a significant shift in Axin1 localization to the membrane fraction in comparison cells expressing only the GFP control. The intensity of Axin1 bands from all membrane, cytoplasmic, and nuclear fractions were quantified across six individual experiments and analysed with a two-way ANOVA with Bonferroni post hoc test. *p < 0.05 (**c**,**d**) Cells transfected with either GFP or FL-C3GFP were exposed to DMEM (control), or Wnt3a for 4 or 24h before being lysed and immunoblotted for phosphorylated Lrp6 (S1490), total Lrp6 and GAPDH. Levels of phosphorylated Lrp6 (normalized to GAPDH) increase >2-fold in GFP-only transfected cells; this increase is significantly abrogated in the presence of g-Pcdh-C3 expression. The intensity of the phospho-Lrp6 band for each condition was normalized to GAPDH in the same lane, and means of 3 individual experiments ± SEM were graphed. A two-way ANOVA with Bonferroni post hoc test was performed. *p < 0.05 MW, molecular weight; kDa, kilodaltons.

In the course of our immunoprecipitation experiments, we noticed that the overall levels of Axin1 were often increased when γ-Pcdh-C3 was overexpressed in comparison to GFP (e.g., compare GFP to C3 lanes in input blots in Fig. 5b,d). We reasoned that this might indicate that interaction with γ-Pcdh-C3 stabilizes Axin1 at the membrane. To test this, we first quantified total endogenous Axin1 levels in HEK293 cells transfected with GFP only vs. γ-Pcdh-A3, -B1, or -C3. Only the C3 isoform significantly increased total Axin1 levels in this assay (Fig. 7a). Subcellular fractionation also demonstrated that cells overexpressing γ-Pcdh-C3 exhibited a significant shift in Axin1 localization to the membrane compared to cells expressing the GFP-only control (Fig. 7b). Together, these data suggest that Axin1 is stabilized when bound to the C3 VCD at the plasma membrane.

### γ-Pcdh-C3 prevents Lrp6 phosphorylation in response to Wnt ligand

The Dvl competition data suggested that γ-Pcdh-C3 might inhibit Wnt signalling at the level of Wnt co-receptor activation, in this case Lrp6 phosphorylation. We addressed this possibility by assaying levels of phospho-Lrp6 (Ser1490) following Wnt3a CM addition in the presence or absence of γ-Pcdh-C3. Indeed, we found that, compared to cells transfected with GFP only, HEK293 cells overexpressing FL C3-GFP had significantly lower induction of Lrp6 phosphorylation at Ser1490 (Fig. 7c,d). Axin1 is required for Lrp6 phosphorylation^57^; γ-Pcdh-C3 could thus inhibit Lrp6 phosphorylation by sequestering a pool of Axin1, making it less available for Dvl-mediated recruitment to activated co-receptor complexes. This stabilized pool of Axin1 may not be utilized for destruction complex assembly (based on our inability to IP GSK3b with the C3VCD; Fig. 6d); it is possible it’s not solely dedicated to Wnt/beta-catenin regulation but rather shunted to other pathways, such as cytoskeletal regulation.

### Evidence that γ-Pcdhs can differentially regulate Wnt signalling in the cerebral cortex *in vivo*

**Figure 8:**
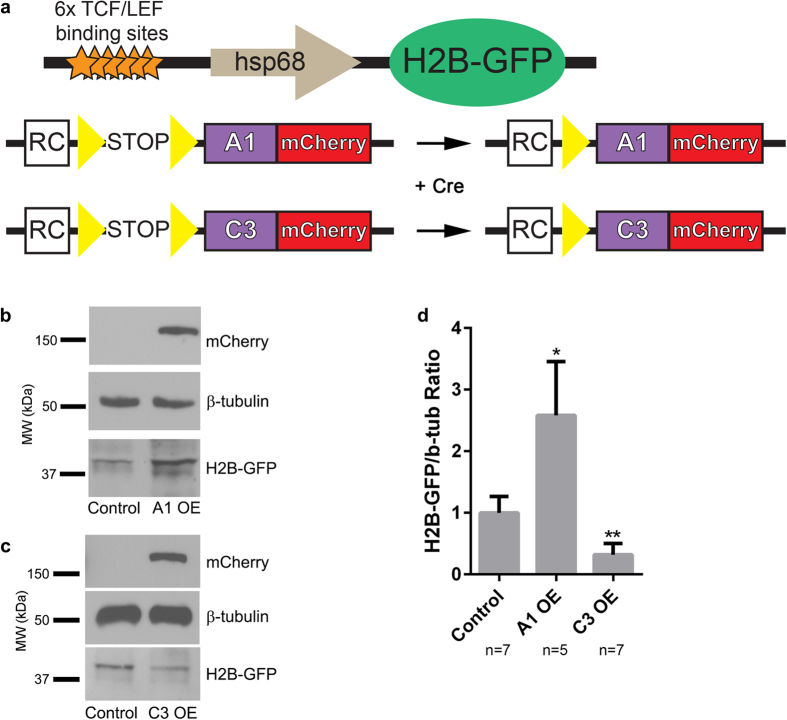
Individual γ-Pcdh isoforms can differentially regulate Wnt signalling in the cerebral cortex *in vivo.* (**a**) Schematic of transgenic mouse alleles utilized. The Wnt reporter allele (top^68^) has 6 TCF/LEF Wnt-responsive binding sites and an hsp68 minimal promoter driving histone 2B-GFP. The A1-mCherry and C3-mCherry transgenes^29^,^32^ are inserted at the ubiquitous Rosa locus. The transgenes are driven by Rosa and CAG elements (RC) only after a floxed stop cassette is excised by Cre. Yellow triangles, loxP sites. (**b**,**c**) Cortical lysates from animals overexpressing γ-Pcdh-A1 or –C3 were used in western blots and probed for GFP to visualize changes in H2B-GFP level, reflecting Wnt signalling activity. Representative Western blot shows an increase in H2B-GFP band intensity in A1-overexpressing animals, while C3-overexpressing animals exhibit a decrease in H2B-GFP band intensity. Blots were probed with anti-mCherry antibody to verify expression of exogenous A1 and C3 isoforms, while β-tubulin is used as a loading control. (**d**) Band intensities of H2B-GFP were normalized to those of β-tubulin in the same lane. Means ± SEM of ≥5 animals were graphed and analysed using one-way ANOVA with Bonferroni post hoc test (to correct for multiple comparisons). *p < 0.05, **p < 0.01. MW, molecular weight; kDa, kilodaltons.

Finally, we asked whether the regulation of Wnt signalling by γ-Pcdhs that we have explored in cultured cells also occurs in the forebrain *in vivo*, where both Wnt and the γ-Pcdhs have important functions^8^,^45^. To answer this question, we crossed a Wnt reporter mouse line, which harbours a transgene containing six TCF/LEF binding sites upstream of a histone 2B (H2B)-GFP fusion^68^ with mice that overexpress, in a Cre-dependent manner, a cDNA encoding either γ-Pcdh-A1-mCherry (predicted to upregulate Wnt signalling; Fig. 1b) or γ-Pcdh-C3-mCherry (predicted to downregulate Wnt signalling; Figs 1, 2, 3)(refs^29^,^32^; Fig. 8a). We activated expression of these γ-Pcdh transgenes in the cerebral cortex by including the *Emx1-Cre* allele^69^. We then prepared cortical protein lysates from these compound transgenics and from littermate controls, blotting for GFP to detect the Wnt-responsive H2B-GFP reporter. Consistent with our *in vitro* data, the cortices of mice overexpressing γ-Pcdh-A1 exhibited a significantly stronger reporter signal (Fig. 8b,d) while those of mice overexpressing γ-Pcdh-C3 exhibited a significantly weaker reporter signal (Fig. 8c,d). This result suggests that, as in HEK293 cells assessed using TOPFlash, distinct γ-Pcdh isoforms can bi-directionally modulate canonical Wnt pathway signalling levels in the cerebral cortex *in vivo*.

## Discussion

In this report, we have demonstrated differential regulation of canonical Wnt signalling by the γ-Pcdhs both *in vitro* and in the cerebral cortex *in vivo*. Prior work by Dallosso *et al*.^13^,^38^ indicated that the *Pcdhg* gene cluster is hypermethylated in both Wilms’ tumour and colorectal cancer, and showed that some γ-Pcdhs could inhibit Wnt signalling and tumour growth. However, few isoforms were tested, and the mechanisms through which γ-Pcdhs might exert an effect on Wnt signalling remained unknown. Using a quantitative TOPFlash assay in HEK293 cells, we tested 20 γ-Pcdh isoforms and found that while the majority of them either have no effect or actually *upregulate* Wnt signalling, γ-Pcdh-C3, uniquely, significantly inhibits Wnt signalling. Focusing on the C3 isoform, we showed that the VCD of C3 is the domain required for its inhibition of the Wnt pathway, and identified Axin1 as a direct binding partner for this domain. The C3 VCD competes with Dvl for binding to Axin1 via its DIX domain, and this C3-Axin1 interaction stabilizes Axin1 at the membrane and leads to reduced phosphorylation of Lrp6. Finally, we show that overexpression of individual γ-Pcdh isoforms *in vivo* can up- or down-regulate a canonical Wnt pathway reporter in the cerebral cortex.

**Figure 9:**
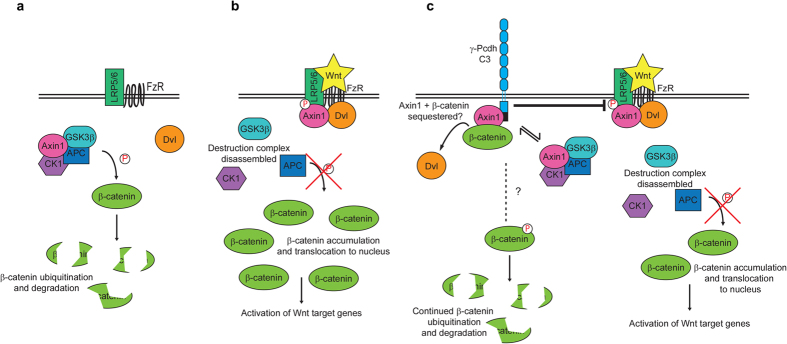
Schematic model of canonical Wnt signalling regulation by γ-Pcdh-C3. Basic ON- (**a**) and OFF- (**b**) states of β-catenin-dependent canonical Wnt signalling. See text for details. (**c**) A model in which γ-Pcdh-C3 competes with Dvl for binding to Axin1; this interaction stabilizes Axin1 at the membrane, and results in reduced phosphorylation of Lrp6 and decreased Wnt signalling. It remains unclear (dashed lines, question marks) whether γ-Pcdh-C3-bound Axin1 can assemble a functional destruction complex, or precisely how β-catenin is regulated by γ-Pcdh-C3.

Our data may indicate a novel mechanism in which interaction of Axin1 with the VCD of γ-Pcdh-C3 sequesters it away from other Wnt pathway components (Fig. 9). Stabilization of Axin1 at the membrane *per se* is sufficient to inhibit Wnt activity in human, *Drosophila,* and *Xenopus* cells^70^,^71^,^72^, contrary to the standard interpretation of the ON-state of Wnt signalling. Activation of canonical Wnt signalling through binding of the Wnt ligand to its receptors activates Dvl, which in turn recruits Axin1/GSK3β complexes to the membrane, promoting the phosphorylation of Lrp6. Phospho-Lrp6 then acts as a competitive inhibitor of GSK3β activity, preventing the phosphorylation and further degradation of β-catenin, as well as of Axin1, effectively activating Wnt signalling^52^,^73^. Because γ-Pcdh-C3 and Dvl interact with the same domain of Axin1, the C-terminal DIX domain as shown here, C3’s competition with Dvl for binding to Axin1 could prevent this downstream activation of Wnt. Consistent with this idea, we show that overexpression of γ-Pcdh-C3 can prevent Lrp6 phosphorylation in response to Wnt. Stabilization and prevention of Axin1 turnover at the membrane by γ-Pcdh-C3 could somehow potentiate or prolong β-catenin degradation and lead to an inhibition of Wnt signalling, since Axin1 is thought to be a limiting component of the destruction complex^74^. However, at this point, it is unclear whether γ-Pcdh-C3-associated Axin1 can assemble a stable destruction complex, as we were able to co-immunoprecipitate β-catenin, but not GSK3β, with γ-Pcdh-C3. In any case, this mechanism of Wnt inhibition—competition with Dvl for binding to Axin1—has some precedence, as another Axin1-binding protein, Axam, was found to inhibit Wnt signalling by preventing the interaction between Dvl and Axin^75^. However, Axam binds to residues 507–712 of Axin1 (within the PP2A/MEKK4 binding site), instead of the DIX domain through which γ-Pcdh-C3 interacts, and is a more potent Wnt/β-catenin antagonist. It was subsequently determined that Axam promotes phosphorylation and degradation of β-catenin (thus inhibiting Wnt signalling) as part of its desumoylation activity^75^. That γ-Pcdh-C3 acts more as a modulator or buffer of Wnt signalling, rather than as a complete inhibitor, is indicated by the fact that a grossly normal cerebral cortex still forms in mice overexpressing this isoform following introduction of *Emx1-Cre* (Fig. 8 and ref. 29). Given the importance of canonical Wnt signalling to cortical morphogenesis^45^, complete inhibition would not be compatible with the normal-sized cortex observed in these mice.

The most striking aspect of the clustered Pcdhs is their diversity. Though analyses of mutant and transgenic mice have revealed a multitude of roles for the γ-Pcdhs in neural development^11^,^25^,^26^,^27^,^28^,^29^,^31^,^32^,^34^,^76^,^77^,^78^,^79^,^80^, we still know relatively little about how individual isoforms differ in their functions and signalling interactions. By testing 20 γ-Pcdh constructs, we found that multiple A and B subfamily isoforms, as well as γ-Pcdh-C5, can actually *upregulate* Wnt signalling in HEK293 cells, as measured by the TOPFlash assay, while γ-Pcdh-C3 is the only isoform that consistently *downregulates* Wnt signalling. Though this is by nature an artificial *in vitro* assay, we confirmed that overexpression of γ-Pcdh-A1 led to an increase in levels of a Wnt reporter protein in the cerebral cortex *in vivo*, just as overexpression of γ-Pcdh-C3 led to a decrease in levels of this reporter (Fig. 8). Though we show that γ-Pcdh-C3 inhibits Wnt signalling by binding to, and stabilizing, Axin1 at the membrane, we did not observe an interaction between other γ-Pcdhs and Axin1. This suggests that there are distinct molecular mechanisms by which some γ-Pcdhs isoforms upregulate Wnt signalling. In this regard, it is important to note the differences in regulation of the *Pcdhg* genes: The A and B subfamily genes are stochastically, monoallelically, and sparsely expressed, while the C isoforms are ubiquitously and biallelically expressed, at least in neurons assayed using single-cell RT-PCR^19^,^21^. It may be that the highly and ubiquitously expressed C3 isoform acts as a general “brake” on overall Wnt signalling levels in all cells, while the expression of distinct combinations of other γ-Pcdhs may modulate signalling levels upward. Given that we know nothing about how homophilic *trans-*interactions or differential *cis-*interactions^22^,^23^ affect the ability of γ-Pcdhs to modulate Wnt signalling, at this point we can only speculate how individual isoforms might collaborate in such a way. The C3 VCD is the site of Axin1 binding and this domain alone is as effective at Wnt inhibition as the FL protein is. This could indicate that Wnt inhibition does not require homophilic *trans-*interactions between γ-Pcdh-C3 ectodomains, but it is equally possible that such interactions cause a conformational change in the protein that enhances Axin1 binding to the VCD. As our experiments with cells expressing FL γ-Pcdh-C3 were conducted in confluent cultures, homophilic *trans-*interactions would have been present. It would be interesting in the future to utilize FL γ-Pcdh-C3 constructs harbouring mutations in EC2/3 predicted (on the basis of recent structural data^22^) to disrupt homophilic interaction to address this possibility.

In addition to its role in Wnt signalling, Axin1 has also been found to interact with synaptic scaffolding molecule (S-SCAM), cyclin-dependent kinase 5 (Cdk5) and Ca2 + /calmodulin-dependent protein kinase II (CaMKII) to play multiple roles in dendrites and dendritic spines^65^,^81^,^82^,^83^. Dishevelled is also known to regulate Rho GTPases and the JNK pathway to affect dendrite morphogenesis^84^. Loss of γ-Pcdhs in the cerebral cortex *in vivo*^28^, or in hippocampal neurons *in vitro*^34^, leads to reduced dendrite arborisation. We recently manipulated γ-Pcdh repertoire in the cortex *in vivo* and found that homophilic interactions between neurons, and between neurons and astrocytes, promote dendrite complexity^29^. Consistent with these data, knockout of CTCF, a major regulator of the Pcdh gene clusters, led to reduced cortical and hippocampal neuron dendrite arborisation^85^. The γ-Pcdhs are also required for normal dendrite self-avoidance in retinal starburst amacrine cells (SAC) and cerebellar Purkinje cells^31^,^32^. The regulation of Wnt signalling by binding and stabilizing Axin1 at the membrane could thus be a novel mechanism through which γ-Pcdh-C3 regulates dendrite development.

In this respect, it will be important to utilize novel mouse models to further delineate the regulation of Wnt signalling by γ-Pcdhs *in vivo.* For example, what is the net effect on Wnt signalling in the *Pcdhg* null mouse lacking all 22 γ-Pcdhs? Given how many γ-Pcdhs were found to upregulate Wnt *in vitro* in this study, one might expect a net decrease in Wnt activity in a null mutant; however, the sole inhibitory γ-Pcdh, C3, is more ubiquitously and more highly expressed than any other isoform, so the opposite result is possible as well. Mice with a constitutive null mutation die at birth^11^, which limits the analyses that can be performed, but a conditional mutant allele^25^,^26^,^28^ could be used to generate tissue-specific knockouts. We are also in the process of generating new lines of mice that either lack one (e.g., a C3 single-isoform mutant) or many (e.g., 8 or 12 of the 22) of the functional variable exons. Given that Wnt pathways in general^84^,^86^ and Axin1 in particular^65^,^83^ can regulate dendrite arborisation and synaptogenesis, it will be important to determine the extent to which the γ-Pcdhs exert their many functions in neural development^25^,^26^,^28^,^30^,^76^,^79^ through Wnt pathway modulation. Similarly, as the *Pcdhg* genes are hypermethylated in a number of cancers^13^,^38^,^39^,^41^ and may act as tumour suppressors, it will be important in the future to develop *in vivo* models of tumour growth and metastasis in mice lacking or overexpressing varying complements of the 22 γ-Pcdh isoforms.

## Methods

### Constructs

Full length γ-Pcdh-GFP constructs were reported in Lobas *et al*.^79^, and full length and truncated HA-tagged γ-Pcdh constructs were reported in Schreiner and Weiner^23^. For VCD constructs, the C3 (or A1 or B1) VCD domain, with or without a 3X nuclear localization signal from SV40 or a palmitoylation signal, were generated by PCR from existing plasmids and cloned into pcDNA3.1 ( + ) with a C-terminal GFP within the ApaI restriction site of the vector. Mouse Axin1 expression constructs were the kind gift of Dr. Nancy Ip. For protein production, mouse Axin(506-832) was amplified from HA-mAxin1 FL and inserted into pGEX-6P-1 (GE Healthcare; GST tagged). The VCD of γ-Pcdh C3 with GFP was amplified from the VCD only constructs and was cloned into pET-24A (Novagen; His-tagged).

### Cell culture

HEK-293 (ATCC® CRL-1573™) cells were maintained in DMEM with 10% fetal bovine serum and penicillin/streptomycin at 37 °C with 5% CO_2_. Wnt3a conditioned media was harvested from LWnt3A (ATCC® CRL2647™) cells as previously described^63^.

### Protein Lysis

Cells were lysed in Mild Lysis Buffer (50 mM Tris-HCl, pH7.4, 150 mM NaCl, 25 mM NaF, 1% TritonX-100). Cell lysates were vortexed for 20 seconds and incubated on ice for 10 minutes and centrifuged at 8000 X g for 5 minutes to pellet cellular debris. The supernatant was collected and used for western blotting and immunoprecipitation.

### Immunoprecipitation

Cell lysates were incubated with 25 μl ThermoFisher Agarose A/G beads and 5 μl of the targeted antibody (antibody concentration of 1 mg/ml) overnight at 4 °C with rotation. Cell lysates were then centrifuged briefly at 2,000 X g for 3 minutes to harvest bead/antibody/protein complex. The beads were washed several times using Mild Lysis Buffer. The protein complexes were eluted by boiling samples for 5 minutes after adding 50 μl of BioRad 2 X Laemmli Buffer.

### Western Blot

Samples were loaded on to a 7.5% Stain-Free TGX Mini Gel and run at 155 V for 58 minutes. The TURBO Mini TGX transfer program on the TransBlot Turbo Transfer System (BioRad) was used to transfer proteins from the gel onto a nitrocellulose membrane. Membranes were blocked for an hour with 5% non-fat milk in TBS-Tween (0.1%) and probed with the primary antibody (usually a 1:1000 dilution in 5% BSA TBS-Tween solution) at 4 °C with rotation. Following 3 5-minute washes in TBST, membranes were probed with host-specific HRP secondary (1:5000 for mouse or rat, 1:1000 for rabbit in 5% non-fat milk in TBS-Tween) for an hour with shaking at RT. Membranes were then washed 5 times with TBST after which the membranes were incubated with SuperSignal West Pico or Femto ECL reagents (ThermoFisher) for signal detection. X-ray films were exposed for a range of times and developed. Films were scanned and analysed in FIJI using the Gel Analyzer plugin.

### Antibodies

The following antibodies were used: mouse anti-GST (Sigma), rat anti-mCherry (Invitrogen), rabbit anti-Axin1 (C76H11) (Cell Signaling Technologies; CST), rabbit anti-Lrp6 (C47E12) (CST), rabbit anti-phospho-Lrp6 (Ser1490) (CST), mouse anti-Myc (9E10) (DSHB), rabbit anti-Myc (71D10) (CST), mouse anti-β-tubulin (Sigma), mouse anti-GAPDH (Abcam), rat anti-HA (Roche), rabbit anti-GFP (Life Technologies), mouse anti-GFP (Roche), chicken anti-GFP (Life Technologies), rabbit anti-FLAG tag (CST).

### Luciferase Assay

Cells were plated to be approximately 75–80% confluent at time of transfection. Transfections for luciferase assays were performed according to manufacturer’s recommendation of 3:1 FuGeneHD to DNA ratio for HEK293 cells, using 0.1 μg of M72 (SuperTOPFlash), 0.1 ng of *Renilla* luciferase and 0.4 μg construct of interest (GFP or individual γ-Pcdh isoforms) per well of a 24-well plate. Other transfections were done following FugeneHD’s protocol database. Cells were incubated with the transfection mixture for 24 hours, after which media were changed to either DMEM (control) or Wnt3a conditioned media (CM). Cells were exposed to Wnt3a CM or DMEM for 24 H before being lysed for Dual Luciferase Reporter Assay (Promega). Cells were lysed using 100 μl 1 x Passive Lysis Buffer on a rocker for 15 minutes. Twenty μl of cell lysates were added to Dual Luciferase Reagent and mixed well through pipetting. Firefly luciferase activity was measured using a Turner Biosystems 20/20 n for 10** **seconds, and 100 μl of Stop & Glo Reagent was added. Tubes were vortexed briefly before assaying for *Renilla* luciferase activity for 10 seconds again. The ratio of the two is referred to as the Relative Luciferase Units. In most cases, data are presented on a log2 scale as a “Wnt Response Index”, which is the fold increase or decrease in the Wnt response seen in cells expressing a γ-Pcdh relative to those expressing a control (GFP only) plasmid.

### Real-Time Quantitative PCR

HEK293 cells plated in 6-well plates were transfected as described above. Cells were incubated with the transfection mixture for 24 hours, after which media were changed to either DMEM (control) or Wnt3a conditioned media (CM). Cells were exposed to Wnt3a CM or DMEM for 24 H before RNA isolation using TRIzol reagent (ThermoFisher) according to manufacturer’s protocol. Two μg of total RNA was used for cDNA synthesis using SuperScript II Reverse Transcriptase (ThermoFisher). The cDNA produced was diluted 1:10 for use in TaqMan® Real Time PCR Assays (ThermoFisher) using a Roche LightCycler 480. The relative expression of genes was analysed using the comparative C_T_ method with the formula: relative expression = 2^−ΔΔC^_T_ (ΔΔC_T_ = ΔC_T_ (Wnt3a) − ΔC_T_ (DMEM), in which ΔC_T_ = C_T_(*AXIN2*) − C_T_(*GAPDH*)). The relative expression of the DMEM control was set to 1.

### Subcellular Fractionation

Samples were harvested and processed 48 hours following transfection with appropriate constructs, according to the Subcellular Protein Fractionation Kit for Cultured Cells’ (ThermoFisher Scientific, 78840) manual.

### Expression of Recombinant Proteins from Bacteria

LB-medium containing the appropriate antibiotics (100 μg/ml ampicillin, 10 μg/ml kanamycin) was inoculated with 10 ml overnight culture per 500 ml medium and shaken at 37 °C until OD_600nm_ between 0.6–0.8 was reached. Expression of protein was induced by addition of IPTG to 200 μM or 500 μM final concentration for pGEX or pET vectors respectively. The culture was further grown at 30 °C for 3 hours. Cells were pelleted by centrifugation at 4000 X g at 4 °C for 20 minutes. Cell pellets were immediately used for protein purification.

### Purification of His_6_-tagged Proteins

The purification of His_6_-tagged proteins was performed using Ni Sepharose 6 Fast Flow (GE Healthcare) The cell pellet was resuspended in 5 ml per 500 ml culture of His binding buffer (50 mM Na_2_H_2_PO_4_, pH 7.4, 300 mM NaCl, 5% glycerol, 10 mM imidazole, 0.1% β-mercaptoethanol, 0.1% Triton-X100) complemented with 1 mg/ml lysozyme and 1 tablet of cOmplete™, Mini, EDTA-free Protease Inhibitor Cocktail. Cell homogenate was incubated on ice for 30 minutes before sonication. Cell lysate was cleared by centrifugation at 5,000 X g for 20 minutes at 4 °C. The supernatant was incubated with 200 μl of 50% Ni Sepharose 6 Fast Flow agarose slurry for an hour at 4 °C with inversion. The agarose was washed 3 times with 5 ml of binding buffer and bound proteins was eluted using 750 μl binding buffer with 250 mM imidazole. Eluted proteins were concentrated and buffer exchanged using an Amicon centrifugal filter unit (Millipore) to PBS before being used in direct binding assays.

### Purification of GST-fusion Proteins

The purification of GST-fusion proteins was performed using Glutathione Sepharose^TM^ 4B (GE Healthcare) The cell pellet was resuspended in 5 ml per 500 ml culture of GST binding buffer (1 X PBS, pH 7.4, 5 mM DTT, 5% glycerol, 0.025% Triton-X100, 10 mM EDTA) complemented with 1 mg/ml lysozyme and 1 tablet of cOmplete™, Mini, EDTA-free Protease Inhibitor Cocktail. Cell homogenate was incubated on ice for 30 minutes before sonication. Cell lysate was cleared by centrifugation at 5,000 X g for 20 minutes at 4 °C. The supernatant was incubated with 200 μl of 50% Glutathione Sepharose^TM^ 4B agarose slurry for an hour at 4 °C with inversion. The agarose was washed 3 times with 5 ml of binding buffer and bound proteins was eluted using 750 μl GST elution buffer (100 mM Tris-HCl, pH8.0, 20 mM reduced glutathione, 100 mM NaCl, 0.025% Triton-X100). Eluted proteins were concentrated and buffer exchanged using an Amicon centrifugal filter unit (Millipore) to PBS before being used in direct binding assays.

### Direct Binding Assay

One μg of His-C3VCD-GFP was mixed with 0.5 μg GST or GST-tagged Axin(506-832) in PBS supplemented with 1 mg/ml BSA and 0.2% NP-40 for 2 hours at 4 °C with inversion. 30 μl of 50% Glutathione Sepharose^TM^ 4B agarose slurry was added to the protein mixture and incubated for an additional hour. The agarose was washed 3 times with PBS supplemented with 0.2% Tween-20 and proteins were eluted using 25 μl GST elution buffer. 2X BioRad Laemmli buffer was added to the eluted proteins and boiled for 5 minutes before use in Western Blot.

### Animals

All animal procedures were approved by the University of Iowa Institutional Animal Care and Use Committee and conformed to all NIH guidelines on the use of animals. The A1-mCherry and C3-mCherry transgenic lines, in which a single γ-Pcdh tagged at the C-terminus with mCherry is expressed from the Rosa locus following Cre excision of a STOP cassette, have been described previously^29^,^32^ and were the kind gift of Julie Lefebvre and Joshua Sanes (Harvard University). The Emx1-Cre (ref. 69; JAX stock #005628) and TCF/Lef:H2B-GFP lines (ref. 68; JAX stock #013752) were obtained from The Jackson Laboratory (Bar Harbor, ME). For all experiments, at least three animals were analysed per genotype.

### Statistical Analyses

Comparisons between constructs, genotypes and treatments were performed by one- or two-way ANOVA using the recommended Bonferroni post-hoc test (corrected for multiple comparisons) in GraphPad Prism. When only two conditions were being compared, a two-way t-test was utilized. Asterisks in figures denote the following significance levels: *p < 0.05; **p < 0.01; ***p < 0.001.

## Additional Information

**How to cite this article**: Mah, K. M. *et al*. The γ-Protocadherin-C3 isoform inhibits canonical Wnt signalling by binding to and stabilizing Axin1 at the membrane. *Sci. Rep.* **6**, 31665; doi: 10.1038/srep31665 (2016).

## Supplementary Material

Supplementary Information
