# Supplementary material for: The γ-Protocadherin-C3 isoform inhibits canonical Wnt signalling by binding to and stabilizing Axin1 at the membrane
Source: Sci Rep. 2016 Aug 17;6:31665. doi: 10.1038/srep31665 (PMC4987702; doi:10.1038/srep31665)
Supplement: Supplementary Information [file srep31665-s1.pdf]

The γ-protocadherin-C3 isoform inhibits canonical Wnt signaling by binding to and stabilizing Axin1 at the membrane

Kar Men Mah[1,2], Douglas W. Houston[1], and Joshua A. Weiner[1,3]*
[1]Department of Biology
[2]Integrated Biology Graduate Program
[3]Department of Psychiatry
The University of Iowa
143 Biology Building
Iowa City, IA 52242
*Corresponding author: joshua-weiner@uiowa.edu

Supplementary Figure S1

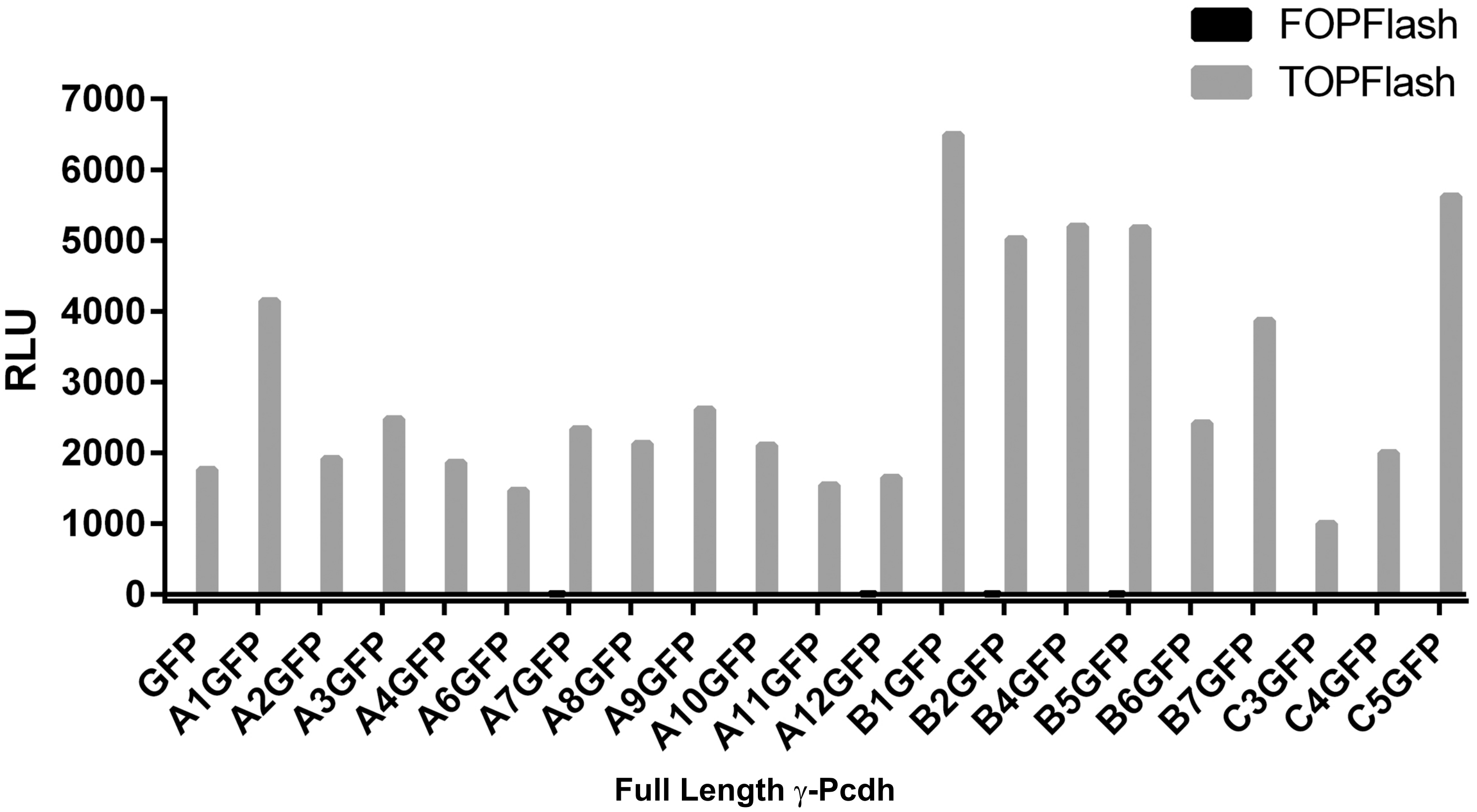

Supplementary Figure S1. Example raw TOPFlash and FOPFlash data for individual γ-Pcdh isoforms. Shown are the Relative Luciferase Units (Firefly/Renilla) for TOPFlash responses 24 hours following Wnt3a CM addition in HEK293 cells transfected with the indicated constructs (gray bars). FOPFlash negative control responses for each condition performed in parallel are shown as black bars; in all cases, these were essentially zero.

Supplementary Figure S2

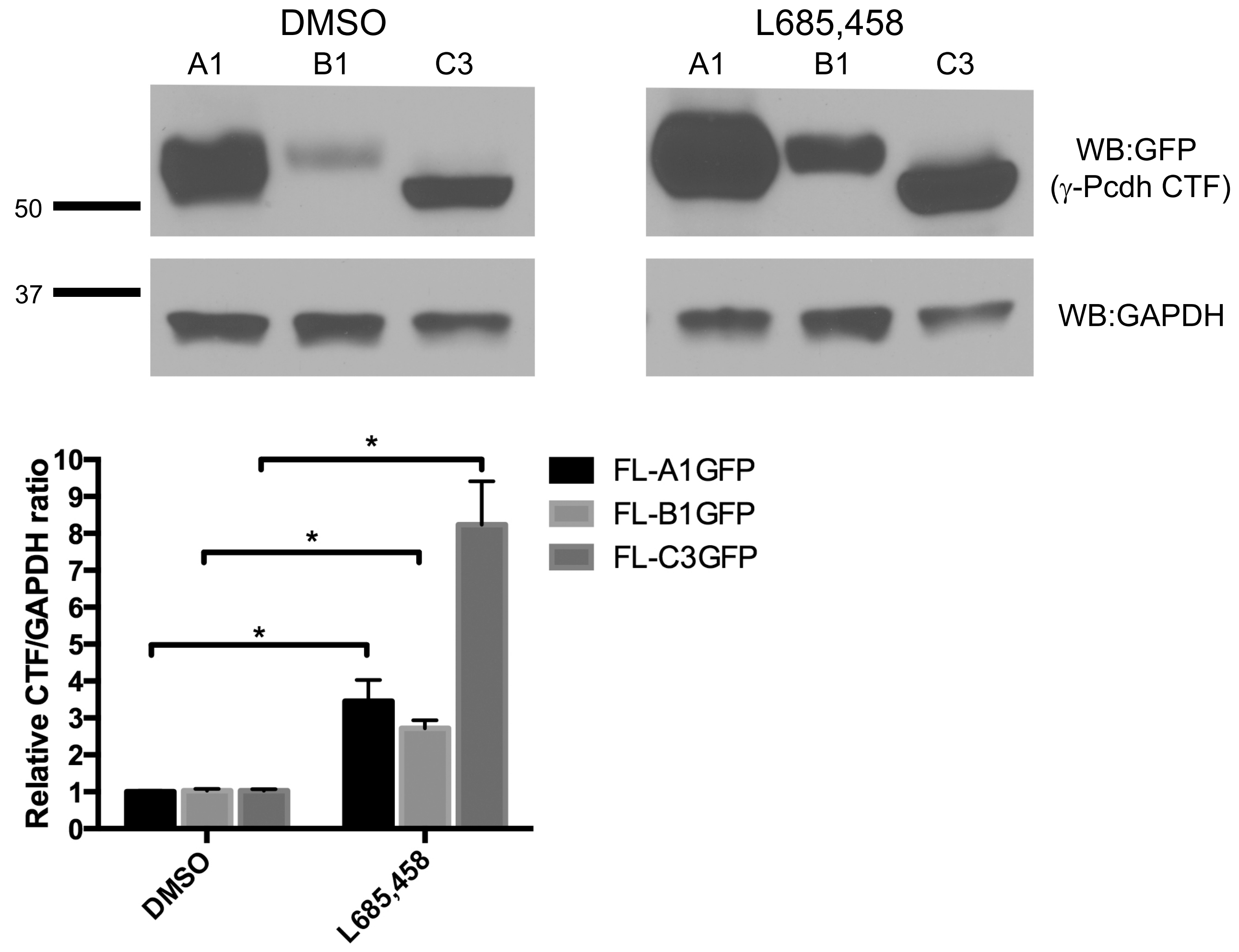

Supplementary Figure S2. Inhibition of γ-Pcdh C-terminal cleavage by the γ-secretase inhibitor L685,458. The ectodomains of γ-Pcdhs can be shed by the activity of matrix metalloproteinases, leaving behind a stub containing the transmembrane domain and the intracellular region (containing both VCD and constant domains). This is then cleaved by γ-secretase, generating a C-terminal fragment (CTF; ~50-60 kDa including a C-terminal GFP tag) that is rapidly degraded (Haas et al., 2005; Hambsch et al., 2005). Treatment of HEK293 cells transfected with FL A1-GFP, B2-GFP, or C3-GFP with the γ-secretase inhibitor L685,458 prevents this cleavage, allowing the transmembrane CTF to build up compared to DMSO (vehicle) treated controls. Graph shows quantification of 3 individual experiments; CTF bands are normalized to GAPDH signal within the same lane. *p<0.05
